# Application of NIR Spectroscopy for the Valorisation of Cork By-Products: A Feasibility Study over the Screening and Discrimination of Chemical Compounds of Interest

**DOI:** 10.3390/ph17020180

**Published:** 2024-01-30

**Authors:** Ricardo N. M. J. Páscoa, Cláudia Pinto, Liliana Rego, Joana Rocha e. Silva, Maria E. Tiritan, Honorina Cidade, Isabel F. Almeida

**Affiliations:** 1 Associated Laboratory for Green Chemistry/Network of Chemistry and Technology, Laboratory of Applied Chemistry, Department of Chemical Sciences, Faculty of Pharmacy, University of Porto, 4050-313 Porto, Portugal; rnpascoa@ff.up.pt; 2 Laboratory of Organic and Pharmaceutical Chemistry, Department of Chemical Sciences, Faculty of Pharmacy, University of Porto, 4050-313 Porto, Portugal; 3 Interdisciplinary Centre of Marine and Environmental Research, University of Porto, Avenida General Norton de Matos, S/N, 4450-208 Matosinhos, Portugal; 4 Applied Molecular Biosciences Unit, MedTech, Laboratory of Pharmaceutical Technology, Department of Drug Sciences, Faculty of Pharmacy, University of Porto, 4050-313 Porto, Portugal; 5 Associate Laboratory i4HB—Institute for Health and Bioeconomy, Faculty of Pharmacy, University of Porto, 4050-313 Porto, Portugal; 6 Dimas & Silva, Lda. Industry, Rua Central de Goda 345, 4535-167 Mozelos, Portugal; 7 Toxicology Research Unit, University Institute of Health Sciences, CESPU, CRL, 4585-116 Gandra, Portugal

**Keywords:** NIR spectroscopy, chemometrics, PLS, PLS-DA, cork residues

## Abstract

*Quercus suber* is considered a sustainable tree mainly due to its outer layer (cork) capacity to regenerate after each harvesting cycle. Cork bark is explored for several application; however, its industrial transformation generates a significant amount of waste. Recently, cork by-products have been studied as a supplier of bioactive ingredients. This work aimed to explore whether near infrared spectroscopy (NIRS), a non-destructive analysis, can be employed as a screening device for selecting cork by-products with higher potential for bioactives extraction. A total of 29 samples of cork extracts were analysed regarding their qualitative composition. Partial least squares (PLS) models were developed for quantification purposes, and R^2^_P_ and RER values of 0.65 and above 4, respectively, were obtained. Discrimination models, performed through PLS-DA, yielded around 80% correct predictions, revealing that four out of five of samples were correctly discriminated, thus revealing that NIR can be successfully applied for screening purposes.

## 1. Introduction

The cork oak, scientifically known as *Quercus suber*, is a tree species that exhibits widespread distribution in the Mediterranean region [1]. The bark of this tree, referred to as cork, is utilized in various industrial applications without posing a threat to the tree. This is attributed to the outer layer’s ability to regenerate after each harvesting cycle [2]. Due to this distinctive property, cork is recognized as a sustainable material.

After harvesting, cork undergoes various industrial treatments involving several production processes, depending on the intended end product. Consequently, different by-products are produced, generating a substantial amount of waste, which is a key worry inherent in this industry [3,4]. One of the most significant by-products is “cork powder”, comprising particles of various shapes and sizes. Global cork production is estimated to reach 200,000 tons, with Portugal being the largest producer at an average of 85,000 tons per year. Approximately 50,000 tons of cork powder are expected to be produced on a global scale each year, taking into account forest productivity, industrial yields, and the quantities of various cork products [3,5].

In recent years, cork bark has emerged as a promising source of sustainable raw materials, capturing industrial interest in harnessing the upcycling potential of cork by-products, such as cork powder, as a rich source of bioactive compounds for various applications [4,6,7].

Aspects like geographic location, cork age (both first and second harvest), age of the tree, and the condition of the planks extracted from the trees are the primary factors influencing cork characteristics [8]. Previous studies have revealed variations in the composition and properties of the different types of cork by-products [4,6]. Consequently, depending on the intended application, a screening tool could be of paramount importance for appropriately selecting a suitable by-product or batch.

When considering the extraction of bioactive ingredients from cork by-products, screening tools can aid in identifying the most suitable batches for specific bioactive extraction, leading to an optimization of the production yield and enhancing the overall sustainability of the process. In this way, batches of by-products with lower bioactive content could be directed towards alternative applications.

Near infrared spectroscopy (NIRS) is a vibrational technique based on the absorption of electromagnetic radiation in the near-infrared (NIR) range, spanning from 13,400 to 4000 cm^−1^. This method provides information about the primary organic chemical components of molecules, including O-H, N-H, and C-H. When coupled with multivariate methods such as principal component analysis (PCA), partial least squares (PLS), and partial least squares discriminant analysis (PLS-DA), this technique becomes a powerful tool for interpreting and analysing spectra [9,10]. The NIR procedure requires the development of an initial calibration, which is then compared with the known chemical composition of the sample. PCA is then typically employed to explore both spectral and analytical data, identifying outliers or unusual samples and evaluating the structure of the dataset [11]. PLS and PLS-DA are commonly applied for quantification and discrimination purposes, respectively [12,13].

NIR is a non-destructive analysis commonly employed in the food and agricultural industries for quality control [14]. More recently, several studies have investigated the application of NIR spectroscopy for the quantification of bioactives in food and plants, demonstrating the potential of this technique [9,10,15]. Additionally, NIRS offers the benefits of being fast, cost-effective, and eco-friendly, requiring small sample sizes and minimal processing [16]. Regarding NIR spectroscopy and cork stoppers, some works have already been developed. In 2010, Prades and co-authors explored the use of NIR for cork plank characterisation in terms of visual quality, porosity, moisture, and geographical origin [17]. The results regarding the geographical origin were better than those obtained for visual quality, porosity, and moisture. The same researcher group validated the good results obtained for geographical origin with NIR spectroscopy using cork stoppers [18]. Later on, in 2014, the same research group applied Vis/NIR spectroscopy for the purpose of predicting the chemical, physical, and mechanical properties of cork stoppers [19]. The best results were obtained for the content of waxes, moisture, and total polyphenols, as well as for density, compression force, and extraction force. Among these, only the results for moisture are applicable for screening purposes. In 2017, NIR spectroscopy was used to perform quality control of cork planks, and the best results was obtained for the colour parameter [20]. More recently, NIR spectroscopy was applied to estimate antioxidant activity (in terms of ABTS and DPPH assays) and total polyphenol content in cork samples [21], yielding coefficient determination results of 0.67, 0.76, and 0.62 for the prediction set, respectively.

The adoption of sustainable processes is already driving the initiatives of industrial organizations. Therefore, effective techniques should be embraced from the inception of a product’s life cycle. This study aims to ascertain whether NIR spectral analysis can serve as a screening tool for the optimized selection of cork by-product samples with higher potential for bioactive extraction. This innovative approach is the first to explore NIR spectroscopy coupled to chemometrics for the analysis of the most valuable chemical compounds present in cork sample by-products, with the aim of fostering the recovery of these compounds. In the end, the reuse of industrial waste produced in large quantities will be enhanced through a cost-effective, rapid, and environmentally friendly technique.

## 2. Results and Discussion

**Figure 1:**
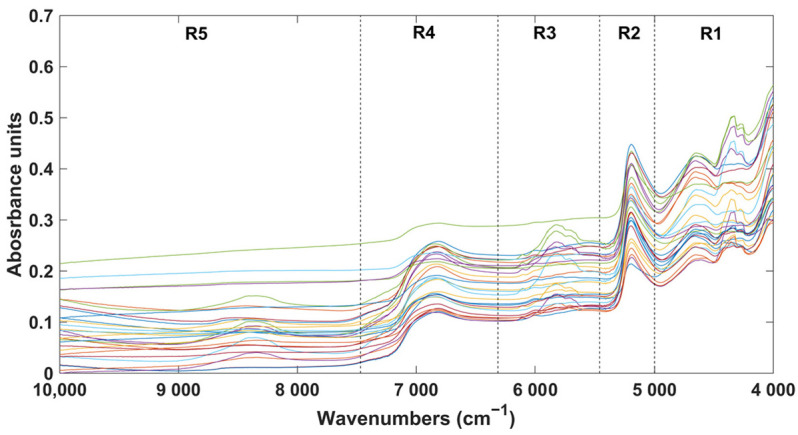
Raw NIR spectra of all samples considering the respective spectral regions. Legend: R1—spectral region R1 (4999 to 4016 cm^−1^); R2—spectral region R2 (5462 to 5003 cm^−1^); R3—spectral region R3 (6311 to 5466 cm^−1^); R4—spectral region R4 (7468 to 6315 cm^−1^); R5—spectral region R5 (9979 to 7472 cm^−1^).

A total of 29 cork powder extract samples were prepared through solid–liquid extraction and analysed using LC-UV, as detailed previously by our group [4,6]. The LC-UV analysis allowed for the quantification of phenolic compounds, namely, gallic acid (1), castalagin (2), protocatechuic acid (3), latifolicinin C acid (4), protocatechuic aldehyde (5), brevifolin carboxylic acid (6), ellagic acid (7), and aesculetin (8). Table 1 presents the amounts of each compound quantified by LC-UV in the 29 samples. Subsequently, these samples were analysed using NIRS (Figure 1).

### 2.1. Spectral Analysis

The NIR spectra with the respective spectral regions are depicted in Figure 1.

As can be observed, the most informative spectral regions were R1 and R3. In spectral region R1, the most relevant bands were identified around 4630 and 4265 cm^−1^, which could be attributed to C-H and C-H_2_ stretching bonds in the combination band region. In spectral region R2, the most important region was centred around 5200 cm^−1^, associated with O-H bonds in the combination region. In spectral region R3, the wavenumbers with the most information were found between 6000 and 5600 cm^−1^, linked to C-H bonds in the first overtone region. Regarding spectral region R4, the most informative bands were within 7200 and 6600 cm^−1^, associated with O-H bonds in the first overtone region [19]. In spectral region R5, no prominent bands were observed. All these bonds are characteristic of the main compounds present in cork, such as cellulose, lignin, and suberin [20].

### 2.2. Quantification through Partial Least Squares (PLS)

Prior to applying PLS, all data (NIR spectra) underwent principal component analysis (PCA). The PCA, utilizing three principal components, captured 92.7% of total variance and revealed no outliers, as well as the formation of clusters. Consequently, all the data were used for further analysis.

As detailed in the Materials and Methods Section 3, specifically in Section 3.5: chemometric analysis, the quantification of these eight parameters was carried out by resorting to PLS. The PLS models were developed using a calibration set (comprising 70% of the data) and were subsequently evaluated for accuracy with an external validation set (comprising the remaining 30% of the data). Table 2 presents the maximum, minimum, median, and average values from both the calibration and validation sets.

As can be observed, the values obtained from the reference procedures (LC-UV) for the validation set fell within the calibration set. However, due to some samples registering below the detection limit of the reference processes for specific chemical parameters, the number of samples varied for each PLS model. These values were not considered.

The optimization of the PLS models indicated that the best processing technique involved using the first derivative of Savitzky–Golay, with a filter width and a polynomial order of 15 points and second order, respectively. The optimal number of latent variables ranged between 4 and 7, depending on the analysed parameter. Concerning the spectral region, the most favourable results were achieved using all the spectra, spectral region R1, and spectral region R3. The values obtained for the calibration and validation of the PLS models are presented in Table 3.

The results indicate that NIR spectroscopy is not capable of accurately determining most of the chemical parameters evaluated in cork residues. Specifically, only the PLS models developed for the quantification of protocatechuic acid yielded good results, with a R^2^_P_ and RER of 0.86 and 6.3, respectively. The poor performance of other models may be attributed to the low sensitivity of NIR spectroscopy. In other words, better results might be achievable if these PLS models were developed using a broader range, especially higher concentrations. However, the majority of the developed PLS models yielded R^2^_P_ values around 0.65, which are considered approximate quantitative predictions [22]. Additionally, most of the developed PLS models had RER values higher than 4, which is acceptable for screening applications [22], aligning with the main purpose of this work. In this context, the obtained results are reasonably acceptable, as this technique appears capable of identifying samples of interest for the recovery of chemical compounds with bioactive properties.

**Figure 2:**
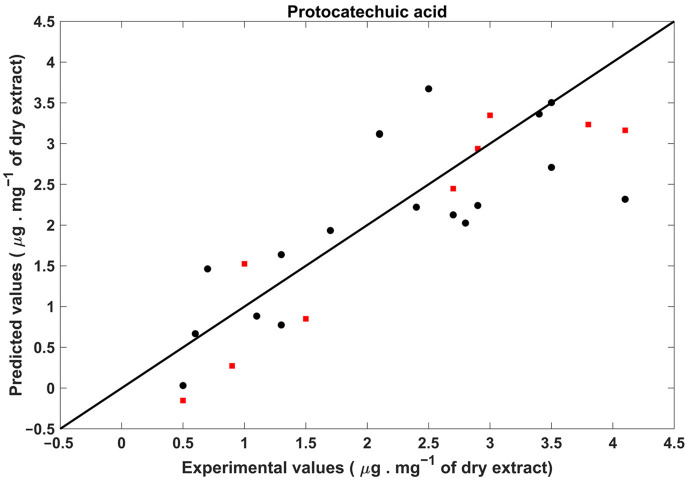
Experimental values against the cross-validation (•) and validation (■) values obtained for protocatechuic acid.

The best PLS model is illustrated in Figure 2.

Regarding the literature, there are no developed works applying NIR spectroscopy to cork sample by-products for the quantification of bioactive compounds. The most similar works have already been cited in the introduction Section 1 and focused on cork stoppers for the quantification of total polyphenols [19] and the antioxidant activities in terms of DPPH and ABTS assays [21]. As observed, the R^2^_P_ values obtained in this work were all higher than 0.61, except for ellagic acid and aesculetin. These values can be compared to R^2^_P_ values obtained for TPC, with R^2^_P_ values of 0.55 and 0.62 in [19] and [21], respectively. Additionally, the R^2^_P_ for ABTS were reported as 0.67 in [21], and for DPPH, it was 0.76 in [21]. It is important to note that the results in this work were obtained for individual compounds and not for a chemical property that encompasses several compounds present in higher quantities that these individual compounds. In this context, the obtained results attest to the suitability of the developed methodology.

**Figure 3:**
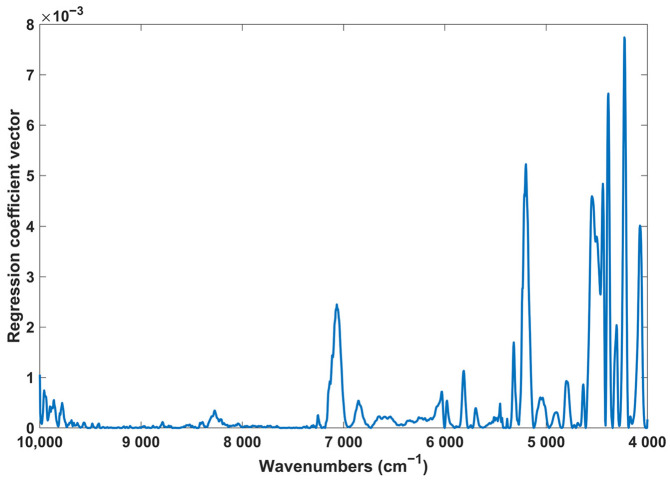
Regression coefficient vector squared for the PLS model of protocatechuic acid.

It is also important to analyse the regression coefficient vectors and verify whether the most important spectral regions are associated with the chemical compounds of interest. Figure 3 displays the regression coefficient vectors obtained for the quantification of protocatechuic acid.

As can be seen, the most important spectral regions for the quantification of protocatechuic acid were identified around 7000 and 5200 and within 4550 and 4050 cm^−1^. The wavenumbers around 7000 and 5200 cm^−1^ can be associated with the first overtone and combination bands of the O-H bonds, while the wavenumbers within 4550 and 4050 cm^−1^ may be related to the combination bands of C-H [23]. Once again, these findings align with the structure of protocatechuic acid, which features several C-H bonds as well as O-H bonds.

The developed PLS models indicate that this technique is suitable for screening purposes, but not for reliable quantifications. It is important to highlight that this technique is rapid, cost-effective, and environmentally friendly without the need for sample preparation. Moreover, this technique can be easily applied in situ at industrial facilities.

### 2.3. Discrimination through PLS-DA

Since one of the reasons pointed out for the lack of accuracy in the developed PLS models was the low sensitivity of NIR spectroscopy, it was decided to develop PLS-DA models to examine whether the method could effectively discriminate cork residue samples with low and high amounts of the studied chemical compounds. As aforementioned, the optimization of the PLS-DA models involved determining the best pre-processing technique, the number of latent variables, and the spectral region using only the calibration set. Regarding the pre-processing technique, the most effective PLS-DA models were established when pre-processing the spectra with Savitzky–Golay using the first derivative and a second polynomial order with a filter width of 15 points, followed by SNV. Concerning the spectral region, the optimal models were achieved by utilising all the spectra or spectral regions R1 and R3, similarly to the PLS models. In relation to the number of latent variables, the developed models utilized between 1 and 3 LV. The validation set was then used for assessing the models’ accuracy through its projection onto the optimized PLS-DA models. The results regarding the percentage of correct predictions, LV, and spectral regions obtained for the validation of the PLS-DA models are shown in Table 4.

The obtained results affirm the suitability of NIR spectroscopy for identifying samples with the highest concentrations of the parameters under study. In fact, the majority of the developed PLS-DA models achieved correct prediction percentages of around 80%, with the PLS-DA model for latifolicinin C acid achieving 100% correct predictions. These outcomes corroborate the PLS results, indicating that this method can effectively be utilised for screening applications.

The analysis of the confusion matrices (Supplementary Materials Figure S1) revealed that two PLS-DA models (latifolicinin C acid and ellagic acid) successfully discriminated samples with the highest amount (category 1) of the respective parameter, achieving an accuracy of 100%. On the other hand, four PLS-DA models achieved 100% correct classifications for discriminating samples with low amounts (category 2) of protocatechuic aldehyde, aesculetin, gallic acid, and latifolicinin C acid. The most challenging misclassifications occurred with the ellagic acid and gallic acid PLS-DA models, particularly with the samples having the lowest and highest amounts, respectively. In both models, only 50% of the samples in each respective category were correctly classified. Overall, all the developed PLS-DA models yielded approximately 80% correct predictions.

**Figure 4:**
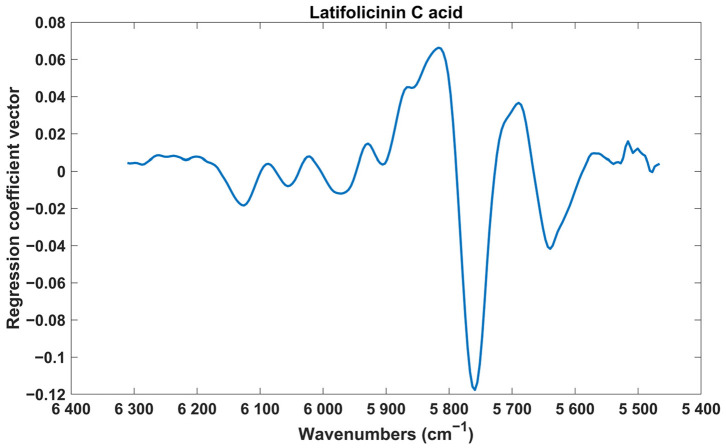
Regression coefficient vector of the PLS-DA model for the discrimination of samples with high and low amounts of latifolicin C acid.

Once again, it is important to analyse the regression coefficient vectors and identify the spectral regions that were most significant for the model. Figure 4 displays the regression coefficient vector obtained for the best PLS-DA model (discrimination of latifolicinin C acid). The regression coefficient vectors of the all the PLS-DA models are provided in Supplementary Materials Figure S2.

The analysis of Figure 4 revealed that the most important wavenumbers were situated around 5750 cm^−1^. This spectral region corresponds to the first overtone region of C-H and C-H_2_ bonds, which aligns with the chemical structure of latifolicinin C acid, containing several of these bonds.

Regarding the analysis of the other regression coefficient vectors (Supplementary Materials Figure S2), the PLS-DA models (gallic acid, protocatechuic acid, protocatechuic aldehyde, and ellagic acid) that utilised the entire NIR spectra demonstrated that the most significant wavenumbers were around 5200 cm^−1^, 7000 cm^−1^, and in the interval of 4900 to 4000 cm^−1^. Meanwhile, the PLS-DA models using spectral region R3 alone (castalagin and brevifolin carboxylic acid) indicated that the most important wavenumbers were within 6000 and 5650 cm^−1^. On the other hand, the PLS-DA model using spectral region R1 and R3 together (aesculetin) revealed the that the most significant wavenumbers were located within 6000 and 5850 cm^−1^ and within 4400 and 4225 cm^−1^. For the PLS-DA models utilising the entire NIR spectra, as mentioned in the PLS analysis, the wavenumbers around 7000 and 5200 cm^−1^ be associated with the first overtone and combination bands of the O-H bonds (which makes sense as all these compounds possess several O-H groups). Simultaneously, the interval within 4900 to 4000 cm^−1^ may be linked to combination bands of C-H (which also makes sense as all these compounds possess several C-H bonds. For the PLS-DA models using spectral region R3 alone (castalagin and brevifolin carboxylic acid) and spectral region R1 and R3 together (aesculetin), the most important wavenumbers belonged to the first overtone region of C-H bonds and the first overtone and combination band region of C-H bonds, respectively [19,20,22,23].

From a global perspective, the developed PLS and PLS-DA models illustrated the suitability of NIR spectroscopy in screening and discriminating cork residue samples. Although the results obtained by the PLS models do not suggest the application of this methodology for accurate quantifications, in practical terms, around 4/5 of samples were correctly discriminated with the PLS-DA models. This enables the further recovery of chemical compounds of interest that are present in these residues.

In this sense, this methodology can foster the development of companies interested in recovering chemical compounds present in cork residue samples, thus promoting a circular economy.

## 3. Materials and Methods

### 3.1. Chemicals

Ethanol was purchased from Honeywell (Charlotte, NC, USA), and ultrapure water was obtained through a Milli-Q^®^ Direct Water Purification System from Millipore (Darmstadt, Germany). HPLC-grade ethanol was sourced from Fisher Chemical (Leicestershire, UK), and formic acid was sourced by Chem Lab NV (Zedelgem, Belgium). Protocatechuic acid, latifolicinin C acid, protocatechuic aldehyde, ellagic acid, and aesculetin were purchased from Aldrich (Saint Louis, MO, USA). Castalagin and brevifolin carboxylic acid were obtained from Phytolab (Vestenbergsgreuth, Germany), and gallic acid was sourced from Acros Organics (Geel, Belgium).

### 3.2. Materials

Cork powders, sourced from Cork Industry Dimas & Silva, Lda, originated from the bark of cork oak (*Quercus suber* L.) harvested in two distinct geographical areas, namely, Portugal and Spain. No pre-treatment procedures were applied. Cork powder extracts were prepared by stirring 5 g of cork powder with H_2_O or 30% EtOH, 50% EtOH, 70% EtOH, 96% EtOH, or EtOH (100 mL) at either room temperature or 40 °C, following the procedure previously reported by our team [4,6], using a magnetic multistirrer (Velp Scientifica, Usmate, Italy) rotating at 700 rpm. The samples were prepared as follows: Samples 1–6 were prepared by stirring cork powder for a period of 2.5 h at room temperature using H_2_O and 30%, 50%, 70%, 96% or 100% EtOH, respectively; samples 7–12 were prepared by stirring cork powder for a period of 2.5 h at 40 °C using H_2_O and 30%, 50%, 70%, 96% or 100% EtOH, respectively; samples 13–18 were prepared by stirring cork powder for a period of 1 h at room temperature using H_2_O and 30%, 50%, 70%, 96% or 100% EtOH, respectively; samples 19–24 were prepared in two extraction cycles by stirring for 1 h in each extraction cycle at room temperature using H_2_O and 30%, 50%, 70%, 96% or 100% EtOH, respectively; and samples 25–29 were prepared in two extraction cycles by stirring for 1 h in each extraction cycle at 40 °C using H_2_O and 30%, 50%, 70% or 96% EtOH, respectively. After extraction, all samples were lyophilised for further analysis through NIR spectroscopy and HPLC.

### 3.3. Liquid Chromatography

A total of 29 samples of cork powder extracts prepared by our team [4,6] were selected for NIRS analysis. The known chemical composition of the samples had been previously assessed via LC-UV based on patterns of compounds commonly reported in the literature for cork extracts, as outlined in our previous work [6]. Each sample was prepared by dissolving in a mixture of H_2_O:EtOH (50:50, *v*:*v*), and subsequently, the solution was passed through a hydrophilic PTFE syringe filter with a pore size of 0.2 μm.

The analytical method was initially established in LC-DAD. For preliminary tests, chromatographic analysis was conducted using a Shimadzu LC-20AD pump equipped with a Shimadzu DGV-20A5 degasser, a Rheodyne 7725i injector fitted with a 20 µL loop, and an SPD-M20A diode array detector (DAD). Data acquisition was performed using Shimadzu LC Lab Solutions software, version 3.50 SP2 (Kyoto, Japan). A commercially available Luna 3 µm PFP (2) obtained from Phenomenex (Torrance, CA, USA) was utilised, with two mobile phases consisting of (A) water:EtOH:formic acid (93.5:5.5:1, *v*:*v*:*v*) and (B) EtOH:formic acid (99:1, *v*:*v*). All solvents were HPLC grade. The chromatographic elution followed a linear gradient: 0–10 min, 100% A; 10–40 min, 100–0% A; 40–50 min, 100% A; followed by re-equilibration of the column before the next run using a flow rate of 0.5 mL min^−1^. The column oven temperature was set at 30 °C, the injection volume was 20 μL, and the detection was performed at 280 nm and at 380 nm. However, the analysis at 280 nm was selected and subsequently employed for both validation and quantification, as it facilitated the identification of the highest number of compounds, as was corroborated by their UV spectrum. Prior to injection, each extract was dissolved in water:EtOH (50:50, *v*:*v*) to achieve a final concentration of 1 mg/mL. The solution was then filtered through a 0.2 μm hydrophilic PTFE syringe filter.

### 3.4. NIR Spectra Acquisition

The NIR spectra of the samples were collected in diffuse reflectance mode using a Fourier-transform near-infrared spectrometer (FTLA 2000, ABB, Dorval, QC, Canada). The spectrometer was equipped with an indium–gallium–arsenide (InGaAs) detector and operated under the control of Bomen–Grams software (version 7, ABB, Dorval, QC, Canada). Each spectrum was derived as the mean of 64 scans, covering the range from 10,000 to 4000 cm^−1^ and obtained with a resolution of 8 cm^−1^. Every sample underwent triplicate analysis, and only the resulting average was utilised for subsequent model analysis. The background was created using a Teflon reference material.

### 3.5. Chemometric Analysis

The chemometric models used in this manuscript were: PCA [24], PLS [12] analysis, and partial least square–discriminant analysis (PLS-DA) [13]. PCA was employed to search for the formation of clusters and to find outliers. The finding of outliers was based on the analysis of the Hotelling’s and squared residual statistics graph. Quantification models, relating the NIR spectra of the samples to the parameters obtained from the reference procedures, namely by HPLC, were developed using PLS. Finally, discrimination models were created through PLS-DA to confirm the ability to distinguish samples with high and low amounts of the analysed chemical parameters. Before the application of these chemometric models, all spectra were previously mean-centred.

The application of PLS and PLS-DA involved optimization in terms of spectral regions, the optimum number of latent variables (LVs), and a pre-processing technique. This optimization was carried out using only the calibration set, indicating that the data were divided into sets: one with 70% for calibration and another with 30% for validation. This division was carried out randomly, ensuring that the values of the chemical parameters analysed in the validation set fell within the values of the calibration set. This was achieved through trial and error until this compromise was obtained. Moreover, for PLS-DA, this division was made while maintaining the same proportion of samples in each category to avoid unbalanced categories [25]. Note that, for PLS-DA, the samples were categorized into two groups: one with chemical values higher than the average value and the other with chemical values lower than the average.

As mentioned earlier, both PLS and PLS-DA were optimized considering only the calibration set. The spectral regions were established considering the influence of water bands (spectral region R2 and R4) on the NIR spectra. Accordingly, the spectra were divided into the following five spectral regions: spectral region R1, spanning from 4999 to 4016 cm^−1^; spectral region R2, covering the range from 5462 to 5003 cm^−1^; spectral region R3, from 6311 to 5466 cm^−1^; spectral region R4, extending from 7468 to 6315 cm^−1^; and spectral region R5, encompassing from 9979 to 7472 cm^−1^. Each of these spectral regions underwent individual testing as well as examination in all conceivable combinations. Various pre-processing techniques, including standard normal variate (SNV) and Savitzky–Golay filtering considering different polynomial orders, filter widths, and the first and second derivatives, were tested to identify the best pre-processing technique. This process was conducted by testing each pre-processing technique individually and in all possible combinations. The SNV is considered a scatter correction pre-processing technique, where all the spectra at each point are subtracted by the average value of the spectrum and then divided by the respective standard deviation [26]. This technique helps to remove baseline shifts of the signal and scales data. The Savitzky–Golay filter is considered a derivative technique that includes a smoothing process. It sequentially finds the derivative of the spectra after selecting the window size and the polynomial degree. This process reduces noise, enhancing signal-to-noise ratio and preserving spectral features while also allowing for the correction of baseline variations [26]. The optimal number of LVs was determined using the leave-one-sample-out cross-validation method, where one sample from the calibration set is excluded during calibration and is then used to evaluate the accuracy of the model. This process is repeated n times, where n represents the total number of samples. Finally, the average of the evaluation is given [25]. For PLS, the optimal calibration models were then determined by balancing between the lowest root mean square error of calibration (RMSEC) and cross-validation (RMSECV), along with the lowest number of LVs. For PLS-DA, the best calibration models were found based on a compromise between the lowest number of LVs and the highest number of correct predictions, which were obtained by summing the diagonal values of the respective confusion matrices. This was performed individually for each chemical parameter.

The evaluation of the optimized models’ accuracy was performed by projecting the validation set onto these models. For PLS models, accuracy was assessed using the coefficient of determination (R^2^_P_), root mean square error of prediction (RMSEP), and range error ratio (RER) parameters. For PLS-DA, accuracy was evaluated in terms of the percentage of correct predictions.

The parameters, RMSEC, RMSECV, and RMSEP, were calculated using the following equation:

(1) $$RMSE=\sqrt{\frac{\sum_{i=1}^{N}\left(\hat{y_{i}}-y_{i}\right)\;}{N}}$$

where $y_{i}$ is the experimental value for sample i; $\hat{y_{i}}$ is the corresponding value obtained for calibration (RMSEC), cross-validation (RMSECV), and prediction (RMSEP); and N is the number of samples.

The RER parameter was calculated according to the following equation:

(2) $$RER=\frac{\left(Y_{max}-Y_{min}\right)}{RMSEP}$$

where $Y_{max}$ and $Y_{min}$ are the maximum and minimum values, respectively, of the validation set.

All the calculations and models were made in the Matlab 2023a environment version 9.14.0.2254940 (MathWorks, Natick, MA, USA), also using the PLS Toolbox version 9.2.1 (Eigenvector Research Inc., Wenatchee, WA, USA).

## 4. Conclusions

As far as we are aware, this represents the first application of NIR spectroscopy in the valorisation of cork residue, specifically aiming to screen and discriminate samples with high amounts of chemical compounds of interest for further extraction.

The developed PLS models for quantification purposes revealed that this methodology can be used for screening application, with R^2^_P_ and RER values around 0.65 and above 4, respectively.

The discrimination models, utilized through PLS-DA, achieved approximately 80% correct predictions, meaning that four out of five samples were correctly discriminated. Notably, the PLS-DA model for the discrimination of latifolicinin C acid yielded 100% correct predictions.

While the obtained results may not enable the application of this methodology in an accurate manner, they do support its use for screening purposes. In this context, the suitability of NIR spectroscopy as a cost-effective, rapid, and environmentally friendly technique for discriminating cork residues with high amounts of chemical compounds of interest was assessed. Additional investigations incorporating a larger sample size across a broader range are necessary to assess the reliability of the developed methodology.

## Figures and Tables

**Table 1:** Concentration of phenolic compounds in cork powder extracts (μg/mg dry extract).

Sample	Gallic Acid (1)	Castalagin (2)	Protocatechuic Acid (3)	Latifolicinin C Acid (4)	Protocatechuic Aldehyde (5)	Brevifolin-Carboxylic Acid (6)	Ellagic Acid (7)	Aesculetin (8)
1	6.4	34.6	2.1	-	0.5	1.7	6.3	1.0
2	9.7	42.9	3.5	1.2	0.5	2.3	13.2	1.2
3	9.8	54.7	3.8	1.4	0.4	3.6	12.2	1.7
4	10.3	48.6	3.4	1.0	0.4	3.6	11.6	1.3
5	7.5	48.1	2.7	2.1	0.2	3.3	17.9	0.6
6	3.0	26.0	1.3	1.6	0.2	2.7	21.6	0.3
7	12.9	42.8	2.8	<LOQ	0.6	1.7	10.2	1.2
8	10.4	48.1	3.1	1.3	0.4	2.2	6.2	1.6
9	11.1	56.5	3.3	1.4	0.4	3.3	12.7	1.5
10	11.4	55.4	3.5	1.5	0.3	3.4	11.5	1.5
11	9.7	47.4	2.9	2.1	0.2	3.2	15.0	1.0
12	6.9	36.0	2.5	1.9	0.2	3.2	20.6	1.0
13	7.7	38.1	2.9	1.0	0.3	2.0	12.6	1.3
14	3.5	43.1	2.4	1.2	0.4	2.7	9.1	1.0
15	4.2	46.3	2.7	1.2	0.4	3.4	12.7	0.8
16	5.0	49.1	3.0	1.1	0.4	3.8	15.5	0.7
17	2.9	31.0	1.7	1.6	0.4	2.7	21.1	0.4
18	1.9	28.2	1.3	1.6	0.3	2.4	36.6	<LOQ
19	6.8	38.4	1.5	<LOQ	0.4	1.7	9.3	1.7
20	2.5	42.4	0.9	1.3	0.4	2.8	21.9	0.7
21	2.1	34.8	0.7	<LOQ	0.3	2.3	25.7	0.6
22	-	36.4	1.0	<LOQ	0.3	2.6	32.9	0.6
23	8.9	38.3	4.1	2.1	0.5	3.0	58.0	1.3
24	4.0	35.4	4.1	1.8	0.5	2.2	77.7	1.0
25	3.1	20.5	0.5	<LOQ	0.5	1.2	5.9	1.9
26	3.6	36.4	0.5	1.0	0.3	2.6	14.7	1.5
27	1.3	27.0	0.6	<LOQ	0.3	1.7	32.5	0.8
28	-	32.9	1.1	1.4	0.4	2.0	29.6	1.0
29	9.7	54.7	2.1	3.1	0.4	3.6	23.5	1.1

Legend: **1–6**: Extracts prepared with 1 × 2.5 h extraction at room temperature using H_2_O, 30%, 50%, 70%, 96%, and 100% EtOH; **7–12**: extracts prepared with 1 × 2.5 h extraction at 40 °C using H_2_O, 30%, 50%, 70%, 96%, and 100% EtOH; **13–18**: extracts prepared with 1 × 1 h extraction at room temperature using H_2_O, 30%, 50%, 70%, 96%, and 100% EtOH; **19–24**: extracts prepared with 2 × 1 h extraction at 40 °C using H_2_O, 30%, 50%, 70%, 96%, and 100% EtOH; **25–29**: extracts prepared with 2 × 1 h extraction at 40 °C using H_2_O, 30%, 50%, 70%, and 96% EtOH.

**Table 2:** Average, standard deviation, minimum and maximum values for the calibration and validation set. The values of minimum, maximum, average, and median are expressed in µg·mg^−1^ of dry sample.

	1	2	3	4	5	6	7	8
Calibration set								
*n*	18	20	18	14	20	20	19	18
Minimum	1.3	20.5	0.5	1.0	0.20	1.2	0.3	5.9
Maximum	12.9	56.5	4.1	3.1	0.60	3.8	1.9	77.7
Median	6.5	40.5	2.3	1.5	0.37	2.7	1.1	20.6
Average	4.2	38.4	2.5	1.4	0.40	2.7	1.0	15.0
**Validation set**								
n	9	9	9	7	9	9	9	9
Minimum	1.9	28.2	0.7	1.0	0.2	1.7	0.6	6.2
Maximum	11.4	55.4	4.1	2.1	0.5	3.6	1.9	29.6
Median	6.4	40.6	2.3	1.4	0.4	2.7	1.1	14.7
Average	7.7	40.6	2.4	1.3	0.4	2.6	1.0	12.2

Legend: 1—gallic acid; 2—castalagin; 3—protocatechuic acid; 4—latifolicinin acid; 5—protocatechuic aldehyde; 6—brevifolin carboxylic acid; 7—ellagic acid; 8—aesculetin; n—number of samples.

**Table 3:** PLS model results, including the ones obtained with the calibration and validation set.

	1	2	3	4	5	6	7	8
Spectral region	all	R3	all	R3	all	R3	all	R1 + R3
LV	6	7	5	6	4	5	7	4
RMSEC	1.3	4.4	0.28	0.22	0.05	0.39	0.13	5.2
RMSECV	3.3	9.2	0.74	0.64	0.10	0.69	0.80	8.0
RMSEP	2.4	5.7	0.57	0.23	0.07	0.46	0.30	5.5
R^2^_C_	0.85	0.81	0.93	0.85	0.75	0.72	0.91	0.64
R^2^_P_	0.61	0.72	0.86	0.63	0.67	0.63	0.42	0.51
RER	3.9	4.8	6.3	3.9	4.2	4.2	3.6	4.2

Legend: 1—gallic acid; 2—castalagin; 3—protocatechuic acid; 4—latifolicinin acid; 5—protocatechuic aldehyde; 6—brevifolincarboxylic acid; 7—ellagic acid; 8—aesculetin; S.D.—standard deviation; n—number of samples; RMSEC—root mean square error of calibration; RMSECV—root mean square error of cross-validation; RMSEP—root mean square error of prediction; RMSEC, RMSECV, and RMSEP are expressed in µg·mg^−1^ of dry extract.

**Table 4:** Percentage of correct predictions considering the validation data set, with the respective number of LV and spectral regions used.

Parameter Analysed	LV	Spectral Region	Percentage of Correct Predictions
Gallic acid	2	All	77.8
Castalagin	3	R3	80.0
Protocatechuic acid	2	All	77.8
Latifolicinin C acid	2	R3	100.0
Protocatechuic aldehyde	1	All	70.0
Brevifolin carboxylic acid	1	R3	80.0
Ellagic acid	3	All	77.8
Aesculetin	3	R1 + R3	80.0

## Data Availability

Dataset available on request from the authors.

## Supplementary Materials

The following supporting information can be downloaded at: https://www.mdpi.com/article/10.3390/ph17020180/s1, Figure S1: Confusion matrices considering only the validation set for the chemical parameters under study. Legend: 1—group with values higher than average; 2—group with values below than average. Figure S2: Regression coefficient vectors of the PLS-DA models for the discrimination of samples with high and low amounts of brevifolincarboxylic acid, castalagin, ellagic acid, gallic acid, latifolicinin C acid, protocatechuic acid, protocatechuic aldehyde, and aesculetin. Table S1. Chemical structures and physicochemical properties of the standards [6,27,28,29,30,31].
