# Supplementary material for: Application of NIR Spectroscopy for the Valorisation of Cork By-Products: A Feasibility Study over the Screening and Discrimination of Chemical Compounds of Interest
Source: Pharmaceuticals (Basel). 2024 Jan 30;17(2):180. doi: 10.3390/ph17020180 (PMC10892220; doi:10.3390/ph17020180)
Supplement: Supplementary file 1 [file pharmaceuticals-17-00180-s001.zip › pharmaceuticals-2748175-supplementary.pdf]

Supplementary Material

Brevifolincarboxylic acid		
Real	Predicted	
	1	2
1	40.0%	10.0%
2	10.0%	40.0%
Total percentage of correct predictions		80.0%

Castalagin		
Real	Predicted	
	1	2
1	40.0%	10.0%
2	10.0%	40.0%
Total percentage of correct predictions		80.0%

Ellagic acid		
Real	Predicted	
	1	2
1	55.6%	0%
2	22.2%	22.2%
Total percentage of correct predictions		77.8%

Gallic acid		
Real	Predicted	
	1	2
1	22.2%	22.2%
2	0%	55.6%
Total percentage of correct predictions		77.8%

Latifolicinin C acid		
Real	Predicted	
	1	2
1	57.1%	0%
2	0%	42.9%
Total percentage of correct predictions		100%

Protocatechuic acid		
Real	Predicted	
	1	2
1	33.3%	11.1%
2	11.1%	44.5%
Total percentage of correct predictions		77.8%

Protocatechuic aldehyde		
Real	Predicted	
	1	2
1	10.0%	30.0%
2	0%	60.0%
Total percentage of correct predictions		70.0%

Aesculetin		
Real	Predicted	
	1	2
1	40.0%	20.0%
2	0%	40.0%
Total percentage of correct predictions		80.0%

Figure S1. Confusion matrices considering only the validation set for the chemical parameters under study. Legend: 1- group with values higher than average; 2- group with values below than average.

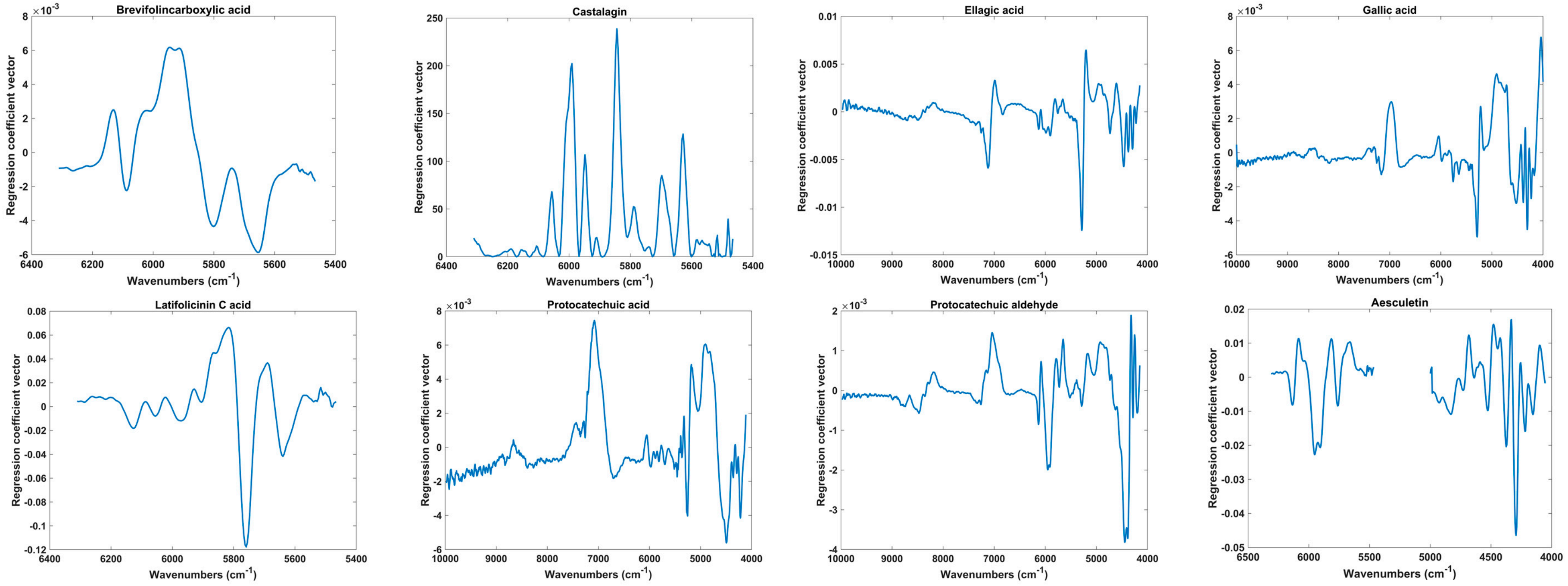

Figure S2. Regression coefficient vectors of the PLS-DA models for the discrimination of samples with high and low amounts of brevifolincarboxylic acid, castalagin, ellagic acid, gallic acid, latifolicinin C acid, protocatechuic acid, protocatechuic aldehyde and aesculetin.

Table S1. Chemical structures and physicochemical properties of the standards [6].

**Compound**	**Structure**	**Molecular weigh**	**Exact mass**	**pKa**	**logP**	**IC_{50} (DPPH)**
Gallic acid (**1**)	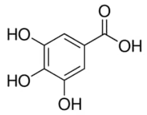	188.13	170.02152	4.21	0.70	8.14 ± 0.26 µM [27]
Castalagin (**2**)	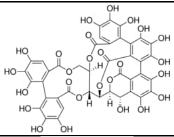	934.64	934.07123	7.14	2.03	15 µM equiv. Trolox [28]
Protocatechuic acid (**3**)	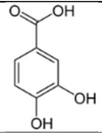	154.12	154.02661	4.26	0.86	147.3 ± 3.6 µM [29]
Latifolicinin C acid (**4**)	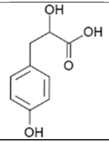	182.17	182.05791	*	*	*
Protocatechuic aldehyde (**5**)	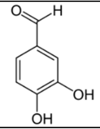	138.12	138.03169	7.46	1.08	74.2 ± 1.2 µM [29]
Brevifolincarboxylic acid (**6**)	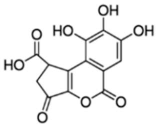	292.2	292.02192	2.91	0.41	4.6 µM [30]
Ellagic acid (**7**)	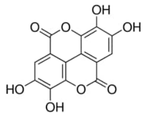	302.19	302.00627	5.54	0.24	4.60 ± 0.15 µM [27]
Aesculetin (**8**)	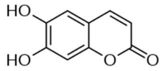	178.14	178.02661	7.91	0.98	25.18 µM [31]

* unreported data. **pKa** and **logP** predicted by Chemaxon for castalagin, protocatechuic aldehyde, brevifolincarboxylic acid, aesculetin and reported in PubChem for gallic acid, protocatechuic acid and ellagic acid.
